# Association of serum cystatin C with white matter abnormalities in patients with amnestic mild cognitive impairment

**DOI:** 10.1111/ggi.13767

**Published:** 2019-09-05

**Authors:** Kentaro Hirao, Fumio Yamashita, Akito Tsugawa, Rieko Haime, Raita Fukasawa, Tomohiko Sato, Misa Okita, Soichiro Shimizu, Hidekazu Kanetaka, Takahiko Umahara, Hirofumi Sakurai, Haruo Hanyu

**Affiliations:** ^1^ Department of Geriatric Medicine Tokyo Medical University Tokyo Japan; ^2^ Department of Ultrahigh Field MRI Institute for Biomedical Sciences, Iwate Medical University Iwate Japan

**Keywords:** cystatin C, deep white matter hyperintensities, mild cognitive impairment, periventricular hyperintensities, white matter hyperintensities

## Abstract

**Aim:**

White matter hyperintensities (WMH) on MRI have been reported to be a risk factor for the conversion from mild cognitive impairment (MCI) to Alzheimer's disease, although the reason remains unclear. In the present study, we hence investigated the associations between WMH volumes and cognitive function, blood levels of various molecules, and the presence of lifestyle‐associated diseases in patients with amnestic MCI.

**Methods:**

The initial data of 38 patients with amnestic MCI and 10 normal control individuals were analyzed. The volumes of periventricular hyperintensities (PVH) and deep WMH (DWMH) were measured on T2 fluid‐attenuated inversion recovery using the imaging software, 3D Slicer; and the association between PVH/DWMH volumes and cognitive function, blood levels of molecules (such as cystatin C [CysC], 25‐hydroxyvitamin D and homocysteine) and the presence of lifestyle‐associated diseases (such as hypertension, hyperlipidemia and diabetes mellitus) were analyzed.

**Results:**

In the MCI group, the PVH volume : intracranial volume ratio significantly correlated with Trail Making Test‐A/B scores and CysC level by Pearson's analysis, and the PVH volume : intracranial volume ratio significantly correlated with only CysC levels, whereas the DWMH volume : intracranial volume ratio did not correlate with any items at all by linear multiple regression analysis.

**Conclusions:**

PVH volume was closely associated with frontal lobe dysfunction, particularly with attention and executive dysfunction. Serum CysC level was associated with PVH volume, which suggests that CysC might be a useful marker for determining treatment strategies for white matter abnormalities in amnestic MCI. **Geriatr Gerontol Int 2019; 19: 1036–1040**.

## Introduction

White matter abnormalities have been recognized to be associated with aging, geriatric depression and dementia, such as Alzheimer disease (AD).^1^, ^2^ Furthermore, white matter hyperintensities (WMH) on magnetic resonance imaging (MRI) have been reported to be a risk factor for the conversion from mild cognitive impairment (MCI) to AD.^3^, ^4^, ^5^ Even among healthy older people, a significant association between WMH and cognitive dysfunction has been reported, although the reason is not clear.^6^, ^7^ Therefore, it is crucial to establish aggressive treatment strategies for the prevention of geriatric syndrome, including dementia and geriatric depression caused by white matter abnormalities. For this, it is very important to assess white matter abnormalities as accurately as possible, and to elucidate the brain mechanisms that are induced by white matter abnormalities from various angles.

Several studies have shown the association between WMH and blood levels of molecules, such as cystatin C (CysC), 25‐hydroxyvitamin D and homocysteine, and the presence of lifestyle‐associated diseases, such as hypertension, hyperlipidemia and diabetes mellitus.^8^, ^9^, ^10^ However, most of these studies have assessed WMH by visual rating, which is not very precise.^3^, ^4^, ^5^ In the present study, we calculated the volume of periventricular hyperintensities (PVH) and deep WMH (DWMH) on T2 fluid‐attenuated inversion recovery (FLAIR) MRI using 3D Slicer, a freely available, open‐source software package for visualization, registration, segmentation, and quantification of medical data (http://www.slicer.org).^11^ Furthermore, we investigated the association between PVH/DWMH volumes and cognitive function, depressive symptoms, blood levels of molecules (such as CysC, 25‐hydroxyvitamin D and homocysteine) and the presence of lifestyle‐associated diseases (such as hypertension, hyperlipidemia and diabetes mellitus) using the data at the initial visit of patients with amnestic MCI.

## Methods

### *Participants*

Outpatients (aged >60 years, <90 years) who were enrolled at the memory clinic or outpatient clinic of Tokyo Medical University, Tokyo, Japan, were prospectively recruited between 2015 and 2018. Written informed consent was obtained from all participants before the study. The study design was approved by the ethics review board of Tokyo Medical University. Initial data of 38 participants with amnestic MCI and 10 normal control (NC) participants, who were spouses of the MCI patients or who were followed at the outpatient clinic but had no memory complaints, were medically stable and had Mini‐Mental State Examination (MMSE) scores of ≥28, were analyzed. All patients underwent detailed general physical, neurological and psychiatric examinations, and extensive laboratory tests, including MRI and single‐photon emission computed tomography (SPECT). SPECT images were analyzed using Neurological Statistical Image Analysis software (Department of Internal Medicine, University of Michigan, Ann Arbor, MI, USA), which are three‐dimensional stereotactic surface projections developed by Minoshima *et al*. for evaluating the spatial distribution of abnormal perfusion to exclude other potential causes of dementia, including dementia with Lewy bodies, frontotemporal lobar degeneration and so on.^12^ The reductions in regional cerebral blood flow of the parietotemporal association cortex on SPECT are recognized as a diagnostic pattern of prodromal AD.^13^

MCI patients were diagnosed as having MCI due to AD according to National Institute on Aging‐Alzheimer's Association criteria, and their MMSE scores were ≥24.^14^ Individuals were excluded from the study if they did not show any reduction in regional cerebral blood flow in the parietotemporal association areas. Individuals were also excluded if they had territorial or cortical infarctions, or if they showed severe white matter disease in which both PVH and DWMH were grade 3 on the Fazekas scale.^15^ Cognitive functions and depressive symptoms were assessed by various neuropsychological tests, such as the MMSE, Frontal Assessment Battery, Trail Making Test (TMT)‐A/B, Wechsler Memory Scale‐Revised‐Logical Memory I, verbal fluency and Geriatric Depression Scale‐15.

### *Laboratory measurements*

Serum CysC, 25‐hydroxyvitamin D and homocysteine levels were measured using colloidal gold agglutination, radioimmunoassay and high‐performance liquid chromatography, respectively. Other laboratory parameters for cerebrovascular risk factors, including total cholesterol, low‐density lipoprotein cholesterol, glucose, hemoglobin A1c, vitamin B_12_, blood urea nitrogen, creatinine, estimated glomerular filtration rate, aspartate transaminase and alanine aminotransferase were measured as well.

### *MRI and volumetric analysis*

Brain MRI scans (3D‐T1 and FLAIR imaging) were carried out using a 1.5‐Tesla scanner (Magnetom; Siemens Medical Systems, Erlangen, Germany) with the following parameters: T1 gradient echo images and FLAIR sequences. FLAIR sequences were obtained with the following parameters: slice thickness 4.0 mm; gap 0.0 mm. For quantitative analysis of WMH volumes, FLAIR images were registered. WMH was defined as the observation of hyperintensity in the white matter area. PVH and DWMH lesions were outlined by a neurologist, using the semi‐automated freeware, 3D Slicer. Furthermore, intracranial volumes (ICV) were calculated using VBM toolbox, which was implemented in Statistical Parametric Mapping (SPM8; Wellcome Institute of Neurology, University College London, London, UK), and the ratio (%) of PVH and DWMH volume to ICV was used for rating white matter abnormalities.

### *Statistical analysis*

Demographic and laboratory data were calculated as the mean ± SD. Statistical analyses were carried out using the Student's *t*‐test, Pearson's test and linear multiple regression analysis. spss 25.0 software (IBM Corporation, Armonk, NY,USA) was used for all statistical analyses.

## Results

**Figure 1:**
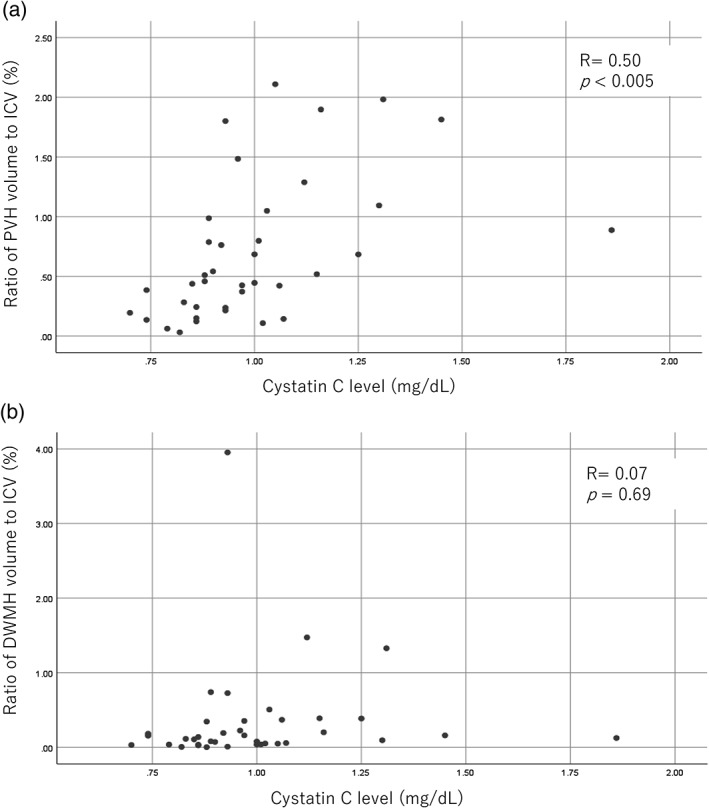
Scatter plots of correlations between cystatin C level and ratios of periventricular hyperintensities (PVH) and deep white matter hyperintensities (DWMH) volumes to intracranial volumes (ICV) in patients with mild cognitive impairment. (a) Ratios of PVH volume to ICV significantly correlated with cystatin C level in patients with mild cognitive impairment. (b) Ratios of DWMH volume to ICV did not significantly correlate with cystatin C levels in patients with mild cognitive impairment. *R* indicates correlation coefficients determined using Pearson's analysis.

A summary of the comparisons of participant characteristics is shown in Table 1. There were significant differences between the MCI group and the NC group with regard to scores on neuropsychological tests, such as MMSE, Frontal Assessment Battery, TMT‐B and Geriatric Depression Scale, whereas both PVH and DWMH volumes in the MCI group were greater than in the NC group, although the differences were not statistically significant. Pearson's analysis showed the correlation of the ratio of PVH and DWMH volume to ICV with continuous variables, including neuropsychological test scores and blood sample data in MCI patients. The PVH volume : ICV ratio significantly correlated with TMT‐A/B and CysC levels. Table 2 shows that in MCI patients, CysC levels correlated significantly and positively with the PVH volume : ICV ratio, but not with the DWMH volume : ICV ratio. Table 3 shows the correlations of the ratio of PVH and DWMH volume to ICV with continuous variables and categorical variables, including the presence or not of hypertension, diabetes mellitus, hyperlipidemia, coronary disease and medications (antidiabetes medication, statins, antithrombotics and antihypertensives) in MCI by linear multiple regression analysis. The PVH volume : ICV ratio significantly correlated with only CysC levels, whereas the DWMH volume : ICV ratio did not correlate with any items at all. Figure 1 shows that the PVH volume : ICV ratio has a statistically significant positive correlation with serum CysC level, whereas the DWMH volume : ICV ratio did not correlate with serum CysC level on Pearson's analysis.

**Table 1:** Demographic, clinical, blood biochemistry and magnetic resonance imaging characteristics of mild cognitive impairment and normal control participants

	MCI (*n* = 38)	NC (*n* = 10)
Sex (male/female)	13/25	5/5
Age (years)	77.4 ± 5.6	76.5 ± 6.2
Education (years)	13.4 ± 2.3	14.4 ± 1.6
MMSE	27.3 ± 1.6*	28.7 ± 0.9
FAB	13.0 ± 2.2*	14.9 ± 2.0
TMT‐A (s)	52.7 ± 20.8	42.2 ± 12.6
TMT‐B (s)	156.9 ± 80.8*	102.5 ± 39.6
WMS‐R‐Logical Memory (immediate)	13.6 ± 6.7	15.5 ± 5.9
VF	14.5 ± 3.4	15.9 ± 3.4
GDS‐15	3.8 ± 3.1*	1.7 ± 1.4
Cystatin C (mg/L)	1.0 ± 0.2	1.1 ± 0.3
25(OH)VitD (ng/mL)	25.0 ± 11.9	19.9 ± 6.1
Homocysteine (nmoL/mL)	10.5 ± 3.5	9.4 ± 2.8
PVH volume (mm^3^)	8905 ± 7678	6175 ± 6253
DWMH volume (mm^3^)	4529 ± 9223	2777 ± 5154
PVH volume ratio (%)	0.70 ± 0.60	0.45 ± 0.46
DWMH volume ratio (%)	0.30 ± 0.70	0.20 ± 0.37
Hypertension, *n* (%)	21 (55)*	2 (20)
Diabetes mellitus, *n* (%)	7 (18)	3 (30)
Hypercholesterolemia, *n* (%)	20 (53)	4 (40)
Coronary artery disease, *n* (%)	3 (8)	1 (10)

25(OH)VitD, 25‐hydroxyvitamin D; DWMH, deep white matter hyperintensities; FAB, Frontal Assessment Battery; GDS, Geriatric Depression Scale; MCI, mild cognitive impairment patients; MMSE, Mini‐Mental State Examination; NC, normal control participants; PVH, periventricular hyperintensities; TMT, Trail Making Test; VF, verbal fluency; WMS‐R‐Logical Memory (immediate), Wechsler Memory Scale‐Revised‐Logical Memory I.

* *P* < 0.05 between the MCI and NC groups.

**Table 2:** Associations between ratio of periventricular hyperintensities and deep white matter hyperintensities volumes to intracranial volumes, neuropsychological test scores and blood sample data in mild cognitive impairment patients

	Coefficient	*P*‐value
	PVH/DWMH	PVH/DWMH
MMSE	−0.18/−0.03	0.29/0.85
FAB	−0.24/−0.09	0.15/0.60
TMT‐A	0.53/0.21	0.001**/0.23
TMT‐ B	0.38/0.13	0.02*/0.44
WMS‐R	−0.06/0.10	0.72/0.54
VF	−0.26/0.02	0.13/0.90
GDS‐15	0.21/−0.10	0.21/0.57
Cystatin C	0.50/0.07	0.002**/0.69
25(OH)VitD	−0.07/−0.01	0.67/0.95
Homocysteine	0.27/−0.17	0.11/0.32

25(OH)VitD, 25‐hydroxyvitamin D; DWMH, deep white matter hyperintensities; FAB, Frontal Assessment Battery; GDS, Geriatric Depression Scale; MCI, mild cognitive impairment patients; MMSE, Mini‐Mental State Examination; NC, normal control participants; PVH, periventricular hyperintensities; TMT, Trail Making Test; VF, Verbal fluency; WMS‐R‐Logical Memory (immediate), Wechsler Memory Scale‐Revised‐Logical Memory I.

* *P* < 0.05, significant correlation between ratio of PVH or DWMH volume to ICV

** *P* < 0.005, significant correlation between ratio of PVH or DWMH volume to ICV.

**Table 3:** Linear regression analyses for factors associated with the ratio of periventricular hyperintensities and deep white matter hyperintensities volume to intracranial volumes in mild cognitive impairment patients

	Ratio of PVH volume			Ratio of DWMH volume		
Independent variable	B	β	*P*‐value	B	β	*P*‐value
Sex	0.143	0.119	0.775	−0.129	−0.231	0.617
Age	0.000	−0.005	0.982	−0.003	−0.065	0.791
AST	0.016	0.338	0.461	0.01	0.451	0.377
ALT	−0.014	−0.209	0.65	−0.02	−0.648	0.219
T‐Cho	−0.003	−0.182	0.743	−0.009	−1.037	0.112
LDL‐Cho	−0.005	−0.236	0.574	0.007	0.664	0.169
BUN	0.000	−0.003	0.992	−0.017	−0.272	0.415
Cr	−1.957	−0.858	0.276	0.85	0.797	0.355
eGFR	−0.03	−0.729	0.134	0.000	0.000	0.999
Glu	−0.002	−0.049	0.887	0.005	0.323	0.402
HbA1c	−0.119	−0.089	0.832	−0.009	−0.014	0.975
VitB12	0.000	−0.18	0.38	0.000	−0.094	0.673
CysC	2.807	1.095	0.016*	0.495	0.413	0.349
25(OH)VitD	−0.013	−0.264	0.238	0.008	0.37	0.143
Homocysteine	−0.05	−0.307	0.378	−0.031	−0.414	0.286
Hypertension	0.83	0.733	0.302	0.09	0.169	0.825
Diabetes mellitus	0.33	0.219	0.588	−0.412	−0.582	0.21
Hypercholesterolemia	−0.401	−0.354	0.351	−0.02	−0.039	0.925
Coronary artery disease	−0.309	−0.125	0.567	0.154	0.134	0.58
Antidiabetic medication	−0.108	−0.044	0.875	0.626	0.543	0.102
Statin	−0.07	−0.055	0.853	0.105	0.177	0.593
Antithrombotics	0.528	0.258	0.297	0.382	0.4	0.155
Antihypertensives	−0.738	−0.653	0.381	−0.2	−0.379	0.641

25(OH)VitD, 25‐hydroxyvitamin D; ALT, alanine aminotransferase; AST, aspartate transaminase; BUN, blood urea nitrogen; Cr, creatinine; CysC, cystatin C; DWMH, deep white matter hyperintensities; FAB, Frontal Assessment Battery; eGFR, estimated glomerular filtration rate; Glu, glucose; HbA1c, hemoglobin A1c; LDL‐Cho, low‐density lipoprotein cholesterol; PVH, periventricular hyperintensities; T‐Cho, total cholesterol; VitB12, vitamin B_12_.

* *P* < 0.05, significant correlation between ratio of PVH or DWMH volume to ICV.

## Discussion

We found that patients with MCI showed greater PVH and DWMH volumes than normal controls, although the difference did not reach statistical significance. Many recent studies, including those carrying out autopsies, have reported the association between cerebrovascular disease and AD.^16^, ^17^ Therefore, the present results that patients with MCI tend to have greater white matter abnormalities compared with NC were consistent with the previous reports. We observed that only the PVH volume : ICV ratio significantly correlated with TMT‐A/B scores in MCI. The association of WMH volume with attention and executive functions in the present study was consistent with the results of previous studies in MCI group.^18^ Furthermore, we observed that attention and executive dysfunction are likely to be associated more closely with PVH volume than DWMH volume in patients with MCI. Although the mechanisms of PVH remain unclear, PVH has been reported to be affected not only by ischemic changes, but also by increased fluid accumulation and reduced integrity of the ventricular ependyma, which might be associated with blood–brain barrier breakdown.^19^ Montagne *et al*. recently reported that both patients with MCI and AD showed blood–brain barrier disruption in the hippocampus and some areas of the cortex and periventricular regions on neuroimaging.^20^ This might be one explanation for MCI patients showing larger PVH volumes, and PVH volumes being more closely associated with cognitive dysfunction compared with NC in the present study. Furthermore, the periventricular region has been reported to include functionally important cholinergic neural pathways, and PVH might exacerbate pre‐existing cholinergic deficits, particularly in AD.^21^, ^22^ The significant association between PVH and cognitive impairment, particularly attention and executive dysfunctions in patients with MCI in the present study, might be caused by cholinergic neural burden by PVH.

We found a significant positive correlation between PVH volume and serum CysC levels. CysC is a cysteine proteinase inhibitor that has been recognized as a marker for early renal impairment, which is more sensitive than creatinine is for estimating glomerular filtration rate.^23^ It has been recognized that kidney dysfunction is a risk factor for white matter abnormalities.^8^, ^24^ Although the association between WMH and kidney dysfunction have not been fully elucidated, particularly regarding subclinical vascular dysfunction, it is assumed that the cells of the kidney and brain are passively perfused throughout systole and diastole by pulsatile flow, whereas their smallest arteries are exposed to high pulsatile pressure by upstream vasodilation.^25^ There was a significant correlation between CysC levels and PVH volumes, but not DWMH volumes. Lee *et al*. recently showed that CysC level significantly correlated with total WMH volume.^26^ We also found a significant association between CysC level and total WMH volume, although we do not show the results. We believe that PVH volume might closely reflect total WMH burden more than DWMH volume, as seen with other studies.^4^, ^27^ However, a significant correlation of CysC with PVH volume, but not with DWMH volume, might simply be owing to a larger range of volumes of PVH than DWMH, because we observed that the volume of PVH was larger than that of DWMH.

Linear regression analysis showed that CysC levels were significantly associated with ratios of PVH volume to ICV, which was independent of creatinine, estimated glomerular filtration rate and other parameters. CysC has been reported to be associated with various cerebrovascular complications, although the mechanisms are not fully understood. Umegae *et al*. reported that CysC is upregulated in regressive astrocytes in a self‐defense response to the process of white matter degeneration in patients with WMH.^28^ Levy *et al*. showed that CysC colocalized with amyloid‐beta in parenchymal and vascular amyloid deposits in AD patient brains.^29^ Furthermore, Sun *et al*. reported that CysC regulates soluble amyloid‐beta levels and is subsequently associated with neuronal deficits in a mouse model of AD.^30^ Taking the aforementioned into account, CysC might have more direct associations with pathological mechanisms of the brain than just renal impairment.

The present study had some limitations. First, the analyses were based on data obtained at one point, namely, at the initial visit. Therefore, we are currently carrying out longitudinal analyses. Second, the number of participants in the NC group was very small. Therefore, it is possible that CysC is associated with white matter abnormalities in the NC group in addition to the MCI group, because the association between CysC and PVH volume were significantly positive in all participants, although we did not show the results. Third, we excluded individuals showing severe white matter diseases, so we should take caution in interpreting the results. Fourth, we did not assess the association between WMH volume of each region and cognitive dysfunction in the present study. Therefore, we are planning to investigate associations between regional WMH volumes and brain function using SPECT in the near future. Fifth, although the underlying pathology in MCI patients was not confirmed in the present study, neuroimaging data were used as part of the diagnostic process. In particular, decreases in regional cerebral blood flow in the parietotemporal association cortex on SPECT is recognized as a diagnostic pattern of MCI due to AD.^13^ Therefore, we feel confident that most cases in the present series of MCI did indeed have AD pathology.

In conclusion, PVH volume is closely associated with frontal lobe dysfunction, particularly with attention and executive dysfunction. Serum CysC level was associated with PVH volume, which suggests that CysC might be a useful marker for determining treatment strategies for white matter abnormalities in amnestic MCI.

## Disclosure statement

The authors declare no conflict of interest.
